# Prevalence of biliary acid malabsorption in patients with chronic diarrhoea of functional characteristics: a prospective study

**DOI:** 10.1186/s12876-021-01637-4

**Published:** 2021-02-09

**Authors:** Virginia Flores, Helena Martínez-Lozano, Federico Bighelli, Javier Orcajo, Javier García-Lledó, Juan Carlos Alonso-Farto, Luis Menchén

**Affiliations:** 1 Servicio de Aparato Digestivo, Hospital General Universitario Gregorio Marañón – Instituto de Investigación Sanitaria Gregorio Marañón, C/ Dr. Esquerdo 46, 28007 Madrid, Spain; 2 Servicio de Medicina Nuclear, Hospital General Universitario Gregorio Marañón – Instituto de Investigación Sanitaria Gregorio Marañón, Madrid, Spain; 3 Departamento de Medicina, Centro de Investigación Biomédica en Red de Enfermedades Hepáticas Y Digestivas (CIBEREHD), Universidad Complutense de Madrid, Madrid, Spain; 4 Centro de Investigación Biomédica en Red de Enfermedades Hepáticas Y Digestivas (CIBEREHD), Madrid, Spain

**Keywords:** Bile acid diarrhoea, Bile acid malabsorption, SeHCAT, Functional diarrhoea, Irritable bowel syndrome, Cholestyramine

## Abstract

**Background:**

Bile acid malabsorption occurs in up to one third of patients with chronic diarrhoea of functional characteristics. The gold standard test for its diagnosis is the ^75^Selenium homocholic acid taurine (^75^SeHCAT) test. The aim of this work is to confirm previous data suggesting that bile acid malabsorption, diagnosed by ^75^Se-HCAT test, is the underlying cause of diarrhoea in a significant proportion of patients previously diagnosed with a functional disorder. In addition, we have analysed the clinical response of bile acid sequestrants in those patients with a bile acid diarrhoea diagnosis.

**Methods:**

This is a prospective, single-centre study including consecutive adult patients diagnosed with chronic diarrhoea of unknown origin and with functional characteristics; systematic rule out of common causes of chronic diarrhoea was performed before bile acid malabsorption evaluation by ^75^SeHCAT scanning. A retention percentage less than 10% was considered positive. Clinical response to cholestyramine was further evaluated in those patients with a positive diagnosis of bile acid diarrhoea

**Results:**

38 patients (20 male, mean age 37.5 years) were finally included. Twenty (52.6%) patients included had a positive ^75^SeHCAT test. Median body mass index was significantly higher in those patients. We did not find significant differences in other clinical or biochemical variables ^75^SeHCAT-positive and ^75^SeHCAT-negative groups. Only 6 of 17 (35.3%) patients responded to cholestyramine treatment; 10 patients did not have response or withdraw the drug due to adverse events. Logistic regression analysis showed that none of the included variables was a predictor of clinical response to cholestyramine.

**Conclusions:**

Bile acid malabsorption occurs in a high proportion of patients suffering from chronic diarrhoea with functional characteristics. Systematic investigation of bile acid malabsorption should be included in the diagnostic algorithms of patients with chronic watery diarrhoea in the routine clinical practice. Absence of response to cholestyramine does not rule out bile acid diarrhoea.

## Background

Chronic diarrhoea can be defined by the presence of more than 3 liquid or soft stools per day and/or a stool volume greater than 200 g per day of liquid or soft consistency, for more than 4 weeks [1–3]. It is a frequent complaint in the primary care setting and a common reason for referral to a gastroenterology clinic, with an estimated prevalence of 5% in Western population [4]. Chronic diarrhoea may result from intestinal inflammatory, malabsorptive, neoplastic or motility disorders, as well as from pancreatic insufficiency or drugs side effects. But in addition, chronic watery diarrhoea may not be related with organic disease, being categorized—according Rome IV criteria of functional digestive disorders—as functional diarrhoea (FD) or diarrhoea-predominant irritable bowel syndrome (IBS-D) [5].

Bile acid malabsorption (BAM) is a well recognised—but often forgotten [6]—pathophysiological event in patients with chronic diarrhoea; indeed, up to a third of patients previously labelled with an IBS-D or FD actually suffer from bile acid diarrhoea (BAD) [7]. This condition can be classified in three types: type 1, secondary to ileal resection or diseases such as Crohn´s; type 2, or “idiopathic”, associated with increased bile acids (BA) production; and type 3, associated with other gastrointestinal conditions that result in BAM, such as cholecystectomy or microscopic colitis [8]. Increased BA entering the colon stimulate water and chloride secretion, inhibit water and sodium absorption and increase colonic motility through several mechanisms revised elsewhere [8, 9]. Type 2 BAD is estimated to occur in 28.1% of patients diagnosed with IBS-D [7], and seems to be the result of an impaired inhibition of BA synthesis due to a decreased levels of fibroblast growth factor 19 (FGF-19) [10]. FGF-19 is an enteroendocrine hormone synthesized in the terminal ileum in response to the stimulation by BA of the nuclear farnesoid X receptor (FXR), expressed by intestinal epithelial cells [11]. Under physiological conditions, FGF-19 is released into the portal circulation and interacts with the FGF receptor 4 on the hepatocytes, decreasing the synthesis of new BA; it also reduces the uptake of BA from the enterocyte, reducing the expression of their transporters [11, 12].

The most widely used tool for BAD diagnosis is the ^75^Selenium homocholic acid taurine (^75^SeHCAT) test, which utilizes a taurine conjugate of 23-selenium-25-homocholic acid given orally [13]; compared with other techniques, it seems to have the highest diagnostic yield [1, 3, 8, 9]. ^75^SeHCAT is absorbed and re-circulated by the same process as natural BAs. A baseline scan by ^75^SeHCAT scintigraphy detects its activity, and a repeat scan is done in 7 days to determine the amount (percentage) of ^75^SeHCAT retained. ^75^SeHCAT retention < 10% suggests moderate BAM and < 5%, severe BAM [8, 9]. Nevertheless, and although its use is recommended for patients with chronic diarrhoea with functional characteristics by several technical reviews and guidelines [1, 3, 14], BAD is still a frequently missed diagnosis. Therefore, the aim of the present work is to confirm that BAM, diagnosed by ^75^Se-HCAT test, is the underlying cause of diarrhoea in a significant proportion of patients previously diagnosed with a functional disorder. In addition, we have analysed the clinical response of BA sequestrants in those patients with a BAD diagnosis.

## Methods

### Patients

We have performed a prospective clinical practice study, single-centre study, with consecutive patients, in order to analyse the prevalence of BAM, assessed by ^75^SeHCAT scanning, in consecutive patients, aged 18 to 75 years, diagnosed with chronic diarrhoea of unknown origin and with functional characteristics, between May, 2016 to June, 2018. Inclusion criteria were as follows: chronic diarrhoea defined by presence of three or more bowel movements per day and/or soft or liquid consistency (Bristol type 6 and 7) for more than 4 weeks, with absence of alarm symptoms [1]; complete colonoscopy and oesophago-gastro-duodenoscopy without macroscopic findings; duodenal and colonic biopsies excluding intraepithelial lymphocytosis/villous atrophy, and microscopic colitis, respectively; negative stool culture, fresh microscopic examination of stool negative for parasites, amoeba serology, and *Clostridium difficile* toxin. Exclusion criteria were: anaemia (Haemoglobin below 12 g/dL), altered thyroid stimulating hormone levels (normal range between 0.35 and 4.94 mU/L), elevated C-reactive protein levels (normal range < 0.5 mg/dL), positive coeliac disease-associated antibodies (anti-transglutaminase IgA or IgG), or decreased faecal elastase levels (normal range above 200 µg/g); diagnosis of diabetes mellitus, primary or secondary immunodeficiency, Parkinson´s or Alzheimer diseases; previous cholecystectomy or intestinal resection; previous treatment with bile acid sequestrants; report of travel to developing countries within the last six months; history of ileitis of unknown cause; previous or on-going diagnosis of chronic pancreatitis; and hypersensitivity to ^75^SeHCAT or any of its components. The use of low doses of loperamide, bulging agents or spasmolytic drugs was not considered exclusion criteria.

The study was approved by the local Ethics Committee and was carried out in accordance with the ethical Declaration of Helsinki in 1964. Informed consent was signed from each patient.

### ^75^SeHCAT scanning protocol

A 370 KBq capsule of ^75^SeHCAT (General Electric Healthcare) was administered orally, with a glass of water, after an overnight fast. Two measurements were then made through a gamma camera equipped with a collimator (GE Discovery MN/CT 670 ES, Low Energy High Resolution [LEHR] collimator) to account for the overall retention of bile acids: three hours (day 0) after administration of the isotope (100% value), and at seven days (day 7). The second measurement is divided by the first to give a proportion of ^75^SeHCAT retained, expressed as a percentage. We considered a positive ^75^SeHCAT test and, thus, diagnosed BAD, at a retention percentage < 10%.

### Follow-up

Treatment with BA sequestrant cholestyramine (Efensol®, powder packets, 3 g per unit) was started at 3 g per day in those patients with a ^75^SeHCAT positive test; in the first visit we explained in writing to the patients the starting dose of cholestyramine and orally how to increase doses up to 12 g per day to achieve symptomatic remission or to reduce doses in case of constipation or other side effects. Patients did not keep a specific diary about stool frequency. Long-term efficacy and safety were analysed in a subsequent programmed visit after 20 ± 4 weeks, in a routine follow-up visit at the outpatient clinic. Clinical response was defined as the relief of symptoms with two or less stools formed or semi-formed daily after the initiation of the treatment. The diagnosis of functional disorder (FD or IBS-D) was made, based on the Rome IV criteria of functional digestive diseases, when the ^75^SeHCAT test was negative.

### Statistical analysis

Continuous variables were expressed as median with interquartile range (IQR) and categorical variables were summarized as number of cases and percentages. To compare continuous variables between the 2 groups (Negative-SeHCAT *vs* Positive-SeHCAT) we used U-Mann Whitney’s test. Chi‐square test or Fisher’s test were used for categorical variables, as appropriate. We performed a logistic regression model to evaluate the independent factors of clinical response to cholestyramine. A p value less than or equal to 0.05 was considered statistically significant. Statistical analysis was performed using SPSS 20.0 (SPSS Inc., Chicago, IL, USA).

## Results

**Fig. 1:**
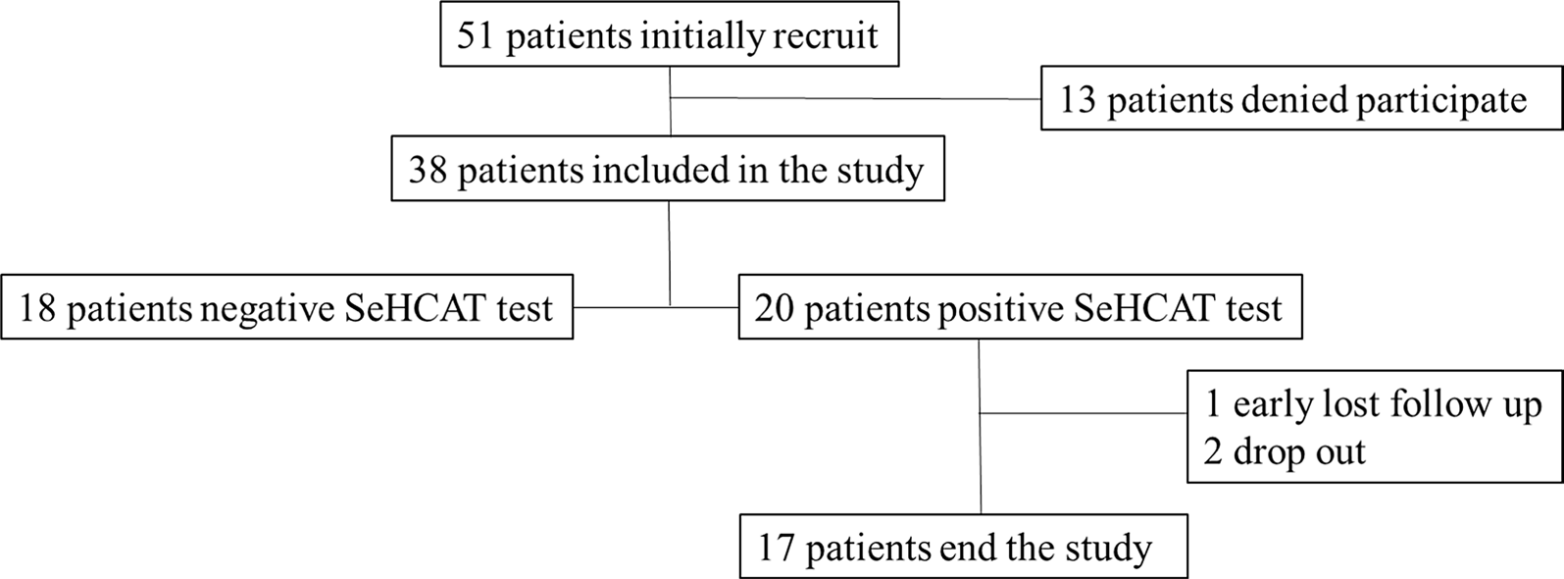
Flow diagram of the study participants

51 consecutive patients with chronic diarrhoea fulfilling inclusion criteria and without exclusion criteria were initially recruited in the study (Fig. 1). 13 patients denied consent for ^75^SeHCAT test; thus, 38 patients (20 male) were finally included. Mean age of the included patients was 37.5 years (IQR 27.2–50.2). Median daily bowel movements at the first visit were 5 (IQR 4–6); 28.9% of patients reported faecal urgency and 48.6%, abdominal pain. We summarized clinical characteristics of the cohort, and concomitant medications in Table 1.

**Table 1:** Patients’ characteristics. Numeric data are expressed as median (IQR: interquartile range). Categorical data are shown as number (%). BMI: Body mass index; PPIs: proton pump inhibitors; Obesity was considered when the BMI was 30.0 or higher

	Overall cohort n = 38	Negative-SeHCAT group n = 18	Positive-SeHCAT group n = 20	*p*-value
Age, years	37.5 (27.2–50.2)	37.0 (23.7–51.7)	39.0 (29.5–49.2)	0.640
Sex, male, %	20 (52.6)	8 (44.4)	12 (60.0)	0.338
Obesity	10 (32.3)	3 (20)	7 (43.8)	0.252
BMI, kg/m^2^	25.1 (21.1–30)	22 (18.7–26.6)	27.8 (23.6–35.3)	**0.006**
Concomitant medications, %	27 (71.1)	12 (66.7)	15 (75.0)	0.572
Antihypertensives	5 (13.2)	1 (5.6)	4 (20.0)	0.344
PPIs	12 (31.6)	7 (38.9)	5 (25.0)	0.358
Statins	3 (7.9)	3 (16.7)	0 (0)	0.097
Spasmolytics	6 (15.8)	4 (22.2)	2 (10.0)	0.395
Probiotics	2 (5.3)	0 (0)	2 (10.0)	0.488
Rifaximin	7 (18.4)	5 (27.8)	2 (10.0)	0.222
Benzodiazepines	2 (5.3)	0 (0)	2 (10.0)	0.488
Antidepressants	3 (7.9)	0 (0)	3 (15.0)	0.232
Anticonvulsants	3 (7.9)	1 (5.6)	2 (10.0)	1.000
Immunosuppressants	2 (5.3)	1 (5.6)	1 (5.0)	1.000
SeHCAT result, % of retention	9.73 (2.9–28.7)	29.1 (21.2–34.1)	3.5 (0.3–6.6)	**0.000**
Symptoms duration before SeHCAT, months	20.8 (12.5 – 46.6)	29.5 (12.6–56.2)	15.3 (12.3–32.3)	0.270
Daily number of bowel movements	5 (4–6)	6 (4–6)	5 (4–7)	0.765
Abdominal pain, %	17 (48.6)	10 (55.6)	7 (41.2)	0.395
Faecal urgency, %	11 (28.9)	6 (40.0)	5 (29.4)	0.529

Bold values indicate statistically significant* p* < 0.05

Twenty (52.6%) out of 38 patients included had a positive ^75^SeHCAT test (median retention 3.5%), whereas 18 patients had a negative result (median retention 29.1%, *p* < 0.001). The median body mass index (BMI) was 27.8 (23.6–35.3) kg/m^2^ in SeHCAT-positive group and 22 (18.7–26.6) kg/m^2^ in SeHCAT-negative group; the difference was statistically significant (*p* = 0.006). We did not find significant differences in age, sex, concomitant medications, symptoms duration, daily number of bowel movements or presence of faecal urgency between SeHCAT-positive and SeHCAT-negative groups (Table 1); no adverse events of the ^75^SeHCAT scanning procedure were reported.

Cholestyramine (starting at 3 g per day and adjusting between 3 g every 48 h and up to 12 g per day, according to the clinical response) was started in 17 patients with positive SeHCAT test. 3 patients refused to start treatment. Regarding effectiveness, 6 patients (35.3%) reported to have symptomatic control of the diarrhoea, with a median daily dose of 6 g; 10 patients did not have response—including two patients under 12 g per day of cholestyramine—or withdrew the drug due to adverse events (AEs); one patient was lost at follow-up (Table 2). Logistic regression analysis showed that none of the included variables was an independent predictor of clinical response to cholestyramine (Table 3).

**Table 2:** Response to cholestyramine. BMI: Body mass index. Obesity was considered when the BMI was 30.0 or higher. Faecal urgency: is defined as the sudden need to rush to empty one's bowels

	No response	Response	*p* value
	N = 10	N = 6	
Age, years	39 (30.5–44.7)	31.5 (20.5–49.2)	0.447
Sex, male, %	7 /70%)	3 (50)	0.607
Obesity BMI ≥ 30, %	4 (50)	0 (0)	0.236
BMI, Kg/m^2^	28.9 (25.7–33.7)	24.2 (23–26.3)	0.540
Abdominal pain, %	5 (50)	0 (0)	0.231
Symptoms duration before SeHCAT, months	16.4 (10.9–26.6)	15.33 (13.8–6)	0.302
Faecal urgency, %	2 (22.2)	2 (33.3)	1.000
Number of stools	5 (3–6)	5 (3–7)	1.000
SeHCAT retention, %	4.5 (1.6–5.6)	3.5 (0.0–9.6)	1.000
Dose of cholestyramine, grams	4.5 (2–7.5)	3 (3–3.7)	0.661
Patients with adverse events, %	4 (40)	3 (50)	1.000

**Table 3:** Logistic regression analysis for factors associated with clinical response to cholestyramine

Variable	OR (CI 95%)	*p* value
Age, years	0.96 (0.87–1.06)	0.426
BMI	0.99 (0.85–1.15)	0.904
Sex, male	2.33 (0.29–18.96)	0.428
Symptoms duration before SeHCAT	1.05 (0.97–1.13)	0.225
Abdominal pain	–	–
Number of stools	0.97 (0.70–1.35)	0.870
Faecal urgency	1.75 (0.17–17.69)	0.635
SeHCAT retention %	1.04 (0.77–1.40)	0.815
Dose of cholestiramine, grams	0.73 (0.37–1.469)	0.383

OR: odds ratio; CI: Confidence interval; BMI: Body mass index. Faecal urgency: is defined as the sudden need to rush to empty one's bowels

Regarding safety profile, 7 of 17 patients experienced AEs, with a total number of 9 adverse effects (Table 2). Constipation was the most frequent AE, affecting to 4 patients; 1 patient reported abdominal pain, and 2 patients reported bloating; 2 patients reported poor tolerability because of a gritty taste. All of AEs were categorized as non-serious.

## Discussion

In this prospective study carried out in a tertiary hospital in Spain, we show that more than 50% of consecutive patients referred for chronic diarrhoea of unknown origin and with functional characteristics were diagnosed with type 2 BAD by means of ^75^SeHCAT test. The results presented herein confirm previous data showing that the prevalence of type 2 BAD is high among patients referred for chronic diarrhoea with functional characteristics [7]. The proportion of patients diagnosed with BAD in our study is similar as previously reported in Spain [15], and even higher than in other series from Northern European countries [16–20]. We must take into account, in addition, that we used a more restrictive criteria for BAM diagnosis (retention percentage less than 10%) than other studies [16, 20], and that patients with previous cholecystectomy or intestinal resection were excluded in our series, on which organic diseases were systematically ruled out by upper gastrointestinal endoscopy and colonoscopy, duodenal and colonic biopsies, faecal microbiological analysis, coeliac disease serology or faecal elastase, among other tests.

Type 2 BAD is associated with increased BA synthesis and excretion [8]. It has been previously described that stool BA concentration correlates with weight, and patients with IBS-D have greater BMI and stool BA concentration than healthy individuals or patients with constipation predominant IBS [21]. Although we did not find statistically significant differences (*p* = 0.25) in the proportion of patients with obesity between those diagnosed with BAD and those with a negative ^75^SeHCAT test result, median BMI was significantly higher in the group of patients with BAD diagnosis included in the present study (*p* = 0.006).

Compared with other methods such as faecal bile acids measurement, measurement of the bile acid intermediate 7α-hydroxy-4-cholesten-3-one (C4) or fibroblast growth factor 19 (FGF19) in serum, or ^14^C-glycocholate breath test, ^75^SeHCAT test seems to have the highest yield for BAD diagnosis [9]; up to date, it is not available in the United States, but its use is increasing in European countries. This molecule is almost exclusively absorbed in the ileum and it is resistant to bacterial degradation; therefore, there are no false positives of this test in patients with bacterial overgrowth. Cut-off level of ^75^SeHCAT retention values varies, among published studies, between < 10% and < 15%, but the 10% cut-off level used in our study may offer greater specificity in terms of predicted response to cholestyramine [22]. Regarding safety, the ^75^SeHCAT capsule contains 370 KBq, with an effective dose of radiation to an adult of 0.26 mSv; for comparative purposes, and as an example, the radiation dose during an abdominal computed tomography (CT) scan is approximately 5.3 mSv [9].

Whether empirical treatment with BA sequestrants is a preferred option than a diagnostic confirmation of BAD by ^75^SeHCAT test is still a matter of debate. Several arguments support the convenience of a definite diagnosis. First, BAD is presumed to be a chronic condition [23], so long-term maintenance treatment is needed; in this sense, the most widely used BA sequestrants such as cholestyramine and colestipol have—besides a particularly disagreeable palatability—frequent side effects such as constipation, bloating and abdominal pain, and it is widely recognized that medication adherence is far from being optimal [24]; therefore, the results of empirical therapy can not be reliably interpreted. Moreover, if BAM diagnosis is confirmed and there is not adequate response or tolerance, dose adjustment can be tried; this issue might be particularly difficult in those patients without a definite diagnosis. Second, empirical treatment might be the first-line option in patients with a higher pre-test probability of a positive diagnosis, like those with Crohn´s disease, previous abdominal radiotherapy, ileal resection or cholecystectomy, but not in those with chronic diarrhoea [25]. And third, it has been recently demonstrated that early BAM diagnosis by means of ^75^SeHCAT test reduces subsequent investigations [26] and healthcare-related costs [27]. Median duration of symptoms until the definitive diagnosis of BAD in our cohort was longer than one year, and it is tempting to speculate that most additional diagnostics tests performed could, presumably, have been avoided using a specific tool for BAD diagnosis earlier. In this sense, the recent update of the British Society of Gastroenterology guidelines for the investigation of chronic diarrhoea, test and treat approach is recommended as opposed to empirical therapy unless no diagnostic test is available [3].

In the present study, we also prospectively assessed the efficacy of bile acid sequestrants in those patients with a BAD diagnosis. Only 6 out of 17 patients achieved symptomatic control. Most of the treated patients included in our study did not respond or withdrew the drug due to AEs. Our results are in line with previous studies [28–30] that show a 40 to 50% response rate to such drugs; although a dose–response relationship—according to the severity of malabsorption—has been described [21], this fact outlines that non-response to treatment with BA sequestrants does not rule out the diagnosis of BAD. In case of response to bile acid sequestrants but poor tolerance, treatment with colesevelam can be considered given its higher affinity for bile acids and better tolerance. However colesevelam is more expensive and more difficult to get access to [30]. Emerging therapeutic treatments such as obeticolic acid are currently not approved for BAM in Spain [31]. Other alternative option is a low-fat diet, which has shown moderate symptomatic improvement [32]. Low doses of loperamide could be used as a complementary treatment with scarce evidence regarding this condition.

Our study has several limitations: first, the sample size is small, and clinical variables associated with a higher pre-test probability of a BAD diagnosis, as well as predictors of response to cholestyramine treatment cannot be found. Second, the patients did not have a specific diary about stool frequency and treatment dose so recall bias could be generated. Third, the results of treatment response should be interpreted with caution since there is not a control group, and its evaluation was made at a single time point. Nevertheless, it is a prospective, real-life setting study on which a systematic investigation of other common causes of chronic diarrhoea was carried out in every patient.

## Conclusion

Our study suggests that BAD—assessed by ^75^Se-HCAT test—occurs in an even higher proportion of patients suffering from chronic diarrhoea with functional characteristis than previously described. Routinely investigation tools for BAM should be widely available, not only in referral centres; its early positioning in the diagnostic algorithms of patients with chronic watery diarrhoea could avoid the performance of more invasive tests or empirical treatment with drugs associated with frequent AEs, since the absence of response to cholestyramine does not rule out BAD. Furthermore, randomized controlled trials assessing different BA sequestrants formulations and doses are needed in this clinical setting.

## Data Availability

The datasets used and/or analysed during the current study are available from the corresponding author on reasonable request.

## Abbreviations

^75^SeHCAT: ^75^Selenium homocholic acid taurine
FD: Functional diarrhoea
IBS-D: Diarrhoea-predominant irritable bowel syndrome
BA: Bile acids
BAM: Bile acids malabsorption
BAD: Bile acids diarrhoea
BMI: Body mass index
AEs: Adverse events
